# Characterizing human postprandial metabolic response using multiway data analysis

**DOI:** 10.1007/s11306-024-02109-y

**Published:** 2024-05-09

**Authors:** Shi Yan, Lu Li, David Horner, Parvaneh Ebrahimi, Bo Chawes, Lars O. Dragsted, Morten A. Rasmussen, Age K. Smilde, Evrim Acar

**Affiliations:** 1 Department of Data Science and Knowledge Discovery, Simula Metropolitan Center for Digital Engineering, Oslo, Norway; 2 COPSAC, Copenhagen Prospective Studies on Asthma in Childhood, Herlev and Gentofte Hospital, University of Copenhagen, Copenhagen, Denmark; 3 Department of Food Science, University of Copenhagen, Copenhagen, Denmark; 4 Department of Nutrition, Exercise and Sports, University of Copenhagen, Copenhagen, Denmark; 5 Swammerdam Institute for Life Sciences, University of Amsterdam, Amsterdam, The Netherlands

**Keywords:** Dynamic metabolomics data, Tensor factorizations, CANDECOMP/PARAFAC, Challenge tests

## Abstract

**Introduction:**

Analysis of time-resolved postprandial metabolomics data can improve our understanding of the human metabolism by revealing similarities and differences in postprandial responses of individuals. Traditional data analysis methods often rely on data summaries or univariate approaches focusing on one metabolite at a time.

**Objectives:**

Our goal is to provide a comprehensive picture in terms of the changes in the human metabolism in response to a meal challenge test, by revealing static and dynamic markers of phenotypes, i.e., subject stratifications, related clusters of metabolites, and their temporal profiles.

**Methods:**

We analyze Nuclear Magnetic Resonance (NMR) spectroscopy measurements of plasma samples collected during a meal challenge test from 299 individuals from the COPSAC_2000_ cohort using a Nightingale NMR panel at the fasting and postprandial states (15, 30, 60, 90, 120, 150, 240 min). We investigate the postprandial dynamics of the metabolism as reflected in the dynamic behaviour of the measured metabolites. The data is arranged as a three-way array: *subjects* by *metabolites* by *time*. We analyze the *fasting state* data to reveal static patterns of subject group differences using principal component analysis (PCA), and *fasting state*-corrected postprandial data using the CANDECOMP/PARAFAC (CP) tensor factorization to reveal dynamic markers of group differences.

**Results:**

Our analysis reveals dynamic markers consisting of certain metabolite groups and their temporal profiles showing differences among males according to their body mass index (BMI) in response to the meal challenge. We also show that certain lipoproteins relate to the group difference differently in the fasting vs. dynamic state. Furthermore, while similar dynamic patterns are observed in males and females, the BMI-related group difference is observed only in males in the dynamic state.

**Conclusion:**

The CP model is an effective approach to analyze time-resolved postprandial metabolomics data, and provides a compact but a comprehensive summary of the postprandial data revealing replicable and interpretable dynamic markers crucial to advance our understanding of changes in the metabolism in response to a meal challenge.

**Supplementary Information:**

The online version contains supplementary material available at 10.1007/s11306-024-02109-y.

## Introduction

Food ingestion triggers many parts of the human metabolism. While the fasting state may reveal certain metabolic differences among individuals, recently *challenge tests* have been used to study also the *postprandial* metabolism of individuals (Bermingham et al., 2023). Analyzing postprandial data may help us understand differences, stratify individuals in terms of their metabolic responses (Bermingham et al., 2023; Harte et al., 2012; Wopereis et al., 2017) and advance personalized nutrition (Berry et al., 2020; Zeevi et al., 2015). For example, postprandial hyperlipidemia, the abnormally increased levels of triglyceride-rich lipoproteins after food intake, is a risk factor for cardiovascular diseases (Botham & Wheeler-Jones, 2013; O’Keefe & Bell, 2007; Poppitt, 2005). Subjects with different body mass index (BMI) values have shown different postprandial responses in terms of amino acids (Bastarrachea et al., 2018; Bondia-Pons et al., 2014) and lipid metabolites (Rämö et al., 2017). Sex differences in insulin sensitivity and metabolome have been observed in the postprandial response (Kumar et al., 2020) (See Lépine et al. (2022) for a recent review on challenge tests and metabolomic responses.) To take it even further, personalized dietary recommendations based on integrating postprandial data with, e.g., gut microbiome characteristics, may lower postprandial glucose levels (Zeevi et al., 2015).

Mining a rich information source such as the postprandial metabolomics data requires advanced data analysis approaches to extract insights. However, there is currently a gap between the complexity of the data and the analysis methods used to analyze such data. The state-of-the-art methods to analyze challenge test data can be considered under *supervised* and *unsupervised* methods. In many cases, there is a study design underlying a challenge test with different groups, e.g., normal vs. hyper-triglycerides (Wojczynski et al., 2011), or healthy vs. diseased or pathological conditions (Kumar et al., 2020). The data in such studies is labelled, and commonly analyzed using *supervised* methods often using univariate methods in which metabolites are analyzed separately, e.g., *t*-tests (LaBarre et al., 2021; Wojczynski et al., 2011) or analysis of variance (ANOVA) models (Berry et al., 2020; Kumar et al., 2020; Rizi et al., 2019; Smilde et al., 2010; Wojczynski et al., 2011) studying group differences. More advanced methods consider explicitly the temporal aspect of the data and/or unbalanced designs, e.g., by using linear mixed models (Müllner et al., 2021), extensions of the analysis of variance-simultaneous component analysis (ASCA) (Erdos et al., 2023). A shortcut for analyzing the temporal behavior is to extract features from the dynamic profiles such as the area under the curve (AUC) (Lairon et al., 2007; Pellis et al., 2012; Rämö et al., 2017). Although these methods are powerful, they rely on labelled data, and may fail to reveal unknown subject stratifications, or they use derived features that do not reveal the full dynamic profile of the data. Univariate methods do not take into account the interplay between metabolites, and may miss the biomarkers by focusing on one metabolite at a time. If there is no a priori group information, e.g., cohort data, which is also the topic of this paper, then such data is unlabelled, and analyzed using *unsupervised* methods, commonly relying on multivariate methods such as principal component analysis (PCA) or clustering. PCA has been used by arranging the data as *subjects-time points* by *metabolites* (Zivkovic et al., 2009) or one time point at a time (Saito et al., 2020). Clustering has been used to cluster metabolites (e.g., averaged across subjects) to create groups of metabolites showing similar time courses (Pellis et al., 2012; Wopereis et al., 2017). These multivariate methods work by considering only summaries of the data or do not utilize the fact that challenge test data has a temporal structure.

**Fig. 1:**
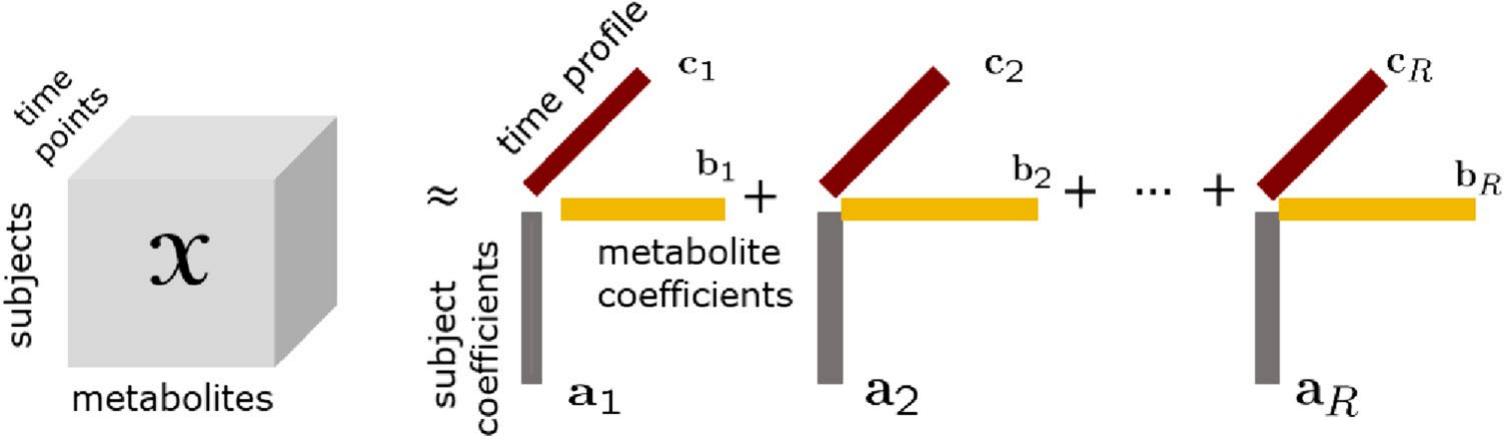
An *R*-component CP model of a third-order tensor with modes: *subjects*, *metabolites*, and *time points*

Postprandial metabolomics data collected at multiple time points from multiple subjects is inherently three-way, and can be arranged as a *subjects* by *metabolites* by *time points* array (see Fig. 1). Multiway data analysis methods (also known as tensor factorizations) (Acar & Yener, 2008; Kolda & Bader, 2009; Smilde et al., 2004) are effective tools for extracting the underlying patterns from such multiway data (also referred to as higher-order tensors), and have been successfully used in data mining, e.g., (Acar et al., 2007; Becker et al., 2023; Williams et al., 2018; Yin et al., 2020).

With the goal of stratifying subjects with no prior labels and understanding differences among subjects based on their metabolic responses to a meal, in this paper, we analyze measurements of plasma samples collected during a meal challenge test from the COPSAC_2000_ cohort (Copenhagen Prospective Studies on Asthma in Childhood) (Bisgaard, 2004). We arrange the metabolomics measurements at several time points from a group of participants as a *subjects* by *metabolites* by *time points* tensor, and use the CANDECOMP/PARAFAC(CP) tensor factorization model (Carroll & Chang, 1970; Harshman, 1970) to reveal the underlying patterns in the data. The CP model summarizes the data by extracting the main patterns of variation in the *subjects*, *metabolites* and *time* modes simultaneously. Extracted patterns are unique under mild conditions (Kolda & Bader, 2009; Kruskal, 1977) and this facilitates interpretation. While CP-based tensor methods have been previously used in longitudinal data analysis, e.g., analysis of gut microbiome (Martino et al., 2021), urine metabolomics (Gardlo et al., 2016), and simulated dynamic metabolomics data (Li et al., 2022), its application to postprandial metabolomics data has been limited. We recently simulated postprandial metabolomics data using a human whole-body metabolic model (Li et al., 2024), and demonstrated that analysis of the T0-corrected data (i.e., the fasting state-corrected data, where the postprandial data is corrected by subtracting the fasting state data) using a CP model together with the analysis of the fasting state data provides a comprehensive picture of the underlying metabolic mechanisms.

The aim of this study is to investigate postprandial dynamics of human metabolism in comparison with the fasting state through the analysis of the time-resolved postprandial metabolomics data collected from the COPSAC_2000_ cohort. We analyze the T0-corrected data using a CP model and the fasting state data using PCA. We demonstrate that the CP model reveals biologically meaningful and replicable patterns (i.e., replicable across subsets of subjects) providing a compact but a comprehensive summary of the postprandial data.

## Data description

The COPSAC_2000_ cohort contains 411 healthy subjects with mothers with a history of asthma^1^. 299 subjects, aged 18 years, attended the food challenge test using a standardized mixed meal (Stroeve et al., 2015) containing 60 g palm olein, 75 g glucose, and 20 g dairy protein in a total volume of 400 ml water-based drink. The size of the drink was made proportional to the daily recommended caloric intake (function of sex, age and height). Blood samples were collected from the participants at eight time points after an overnight fasting, i.e., at the fasting state, and 0.25, 0.5, 1, 1.5, 2, 2.5, and 4 h after the meal intake. The samples were put on ice, and within 4 h split into EDTA plasma before being stored at $-80 ^{\circ }$C.

Plasma samples were measured using NMR (nuclear magnetic resonance) spectroscopy through Nightingale Blood Biomarker Analysis, which provides 250 features.^2^ These features consist of lipoproteins, apolipoproteins, aminoacids, fatty acids, glycolysis-related metabolites, ketone bodies and an inflammation marker. Lipoproteins consist of four main lipoprotein classes: HDL (high density lipoprotein), IDL (intermediate density lipoprotein), LDL (low density lipoprotein), and VLDL (very low density lipoprotein), and subclasses divided according to particle sizes: XXL (extremely large), XL (very large), L (large), M (medium), S (small) and XS (very small). Cholesterol (C), cholesterol ester (CE), free cholesterol (FC), triglyceride (TG), phospholipid (PL) levels of these classes and subclasses are among the list of metabolites. In addition, we included measurements of insulin and C-peptide. The total number of features we included in this study is 161, and these features are given in Table S1.1 in Supplementary Material S1.

Seven subjects are removed before the analysis since two subjects have a large amount of missing data, four subjects have extremely high levels of acetate, and one subject is detected as an outlier for the CP model (for outlier detection, see (Bro, 1997)). In total, there are 140 male and 152 female subjects included in the analysis.

The T0-corrected metabolomics data, i.e., the postprandial data corrected by subtracting the fasting state data, is arranged as a third-order tensor ${\varvec{\mathscr{X}}}$ of size $I\times J \times K$, where *I*, *J*, *K* denotes the number of subjects (140 males or 152 females), metabolites (161), and time points (7 time points, i.e., fasting state data is subtracted from other time points and excluded in T0-corrected data), respectively. The fasting state data is arranged as a matrix of size $I\times J$.

In addition to the metabolomics data, additional information such as different body composition, and insulin resistance measures were obtained from the subjects. Weight, height, and waist circumference were directly measured, and consequently BMI and waist/height ratio were calculated. Body composition was estimated by bioelectrical impedance using a Tanita scale (TANITA MC-780MA), to obtain body muscle mass and fat mass, and subsequently, body fat percentage, muscle to fat ratio, fat mass index (fat mass/height^2^ (kg/m^2^)), and fat free mass index ((muscle mass/2.2) ×﻿ 2.20462/height^2^ (kg/m^2^)). Insulin resistance was assessed using HOMA-IR (Homeostatic Model Assessment for Insulin Resistance), by the formula: fasting insulin (microU/L) × fasting glucose (nmol/L) / 22.5. Descriptive statistics on these variables stratified by sex are provided in Table S1.2 in Supplementary Material S1.

## Methods

### CP model

The CP model (Carroll & Chang, 1970; Harshman, 1970; Hitchcock, 1927) approximates a higher-order tensor as the sum of a minimum number of rank-one tensors as in Fig. 1. An *R*-component CP model of a third-order tensor ${\varvec{\mathscr{X}}} \in {\mathbb{R}}^{I \times J \times K}$ with modes: *subjects*, *metabolites*, and *time points* represents the data as follows:

1 $$\begin{aligned} {\varvec{\mathscr{X}}} \approx \sum _{r=1}^{R} {\varvec{\mathbf {\MakeLowercase {a}}}}_{r}\circ {\varvec{\mathbf {\MakeLowercase {b}}}}_{r} \circ {\varvec{\mathbf {\MakeLowercase {c}}}}_{r} = \llbracket {\varvec{\mathbf {\MakeUppercase {A}}}}, {\varvec{\mathbf {\MakeUppercase {B}}}}, {\varvec{\mathbf {\MakeUppercase {C}}}} \rrbracket , \end{aligned}$$

where $\circ$ denotes the vector outer product; ${\varvec{\mathbf {\MakeLowercase {a}}}}_{r}$, ${\varvec{\mathbf {\MakeLowercase {b}}}}_{r}$, ${\varvec{\mathbf {\MakeLowercase {c}}}}_{r}$ corresponds to the *r*th column of factor matrices ${\varvec{\mathbf {\MakeUppercase {A}}}}\in {\mathbb {R}}^{I\times R}, {\varvec{\mathbf {\MakeUppercase {B}}}}\in {\mathbb {R}}^{J\times R}$, and ${\varvec{\mathbf {\MakeUppercase {C}}}}\in {\mathbb {R}}^{K\times R}$, respectively. Each component $({\varvec{\mathbf {\MakeLowercase {a}}}}_{r},{\varvec{\mathbf {\MakeLowercase {b}}}}_{r},{\varvec{\mathbf {\MakeLowercase {c}}}}_{r})$ may reveal subject groups (in ${\varvec{\mathbf {\MakeLowercase {a}}}}_{r}$), groups of metabolites (in ${\varvec{\mathbf {\MakeLowercase {b}}}}_{r}$) related to those subject groups following a specific temporal profile (given by ${\varvec{\mathbf {\MakeLowercase {c}}}}_{r}$). The CP model is unique up to permutation and scaling ambiguities under mild conditions (Kolda & Bader, 2009). Permutation ambiguity indicates that the order of rank-one components is arbitrary while the scaling ambiguity corresponds to arbitrarily scaling each vector in component *r*, i.e., (${\varvec{\mathbf {\MakeLowercase {a}}}}_{r}$, ${\varvec{\mathbf {\MakeLowercase {b}}}}_{r}$, ${\varvec{\mathbf {\MakeLowercase {c}}}}_{r}$), as long as the product of the norms of the vectors stays the same.

The uniqueness of the CP model without additional constraints on the extracted patterns is an advantage compared to other types of tensor factorizations such as the Tucker decomposition (Tucker, 1966). The uniqueness facilitates the interpretability of the CP components, i.e., when the model is unique, each component $({\varvec{\mathbf {\MakeLowercase {a}}}}_{r},{\varvec{\mathbf {\MakeLowercase {b}}}}_{r},{\varvec{\mathbf {\MakeLowercase {c}}}}_{r})$ can be interpreted one at a time potentially revealing biomarkers for a specific stratification of subjects. The Tucker decomposition is a more flexible model than the CP model and it is not unique in terms of extracted patterns. Additional constraints on the factor matrices and core array in the Tucker decomposition can achieve uniqueness (Kolda & Bader, 2009). For instance, higher-order singular value decomposition (HOSVD) with orthogonality constraints on all factor matrices as well as all-orthogonal core can help with uniqueness. HOSVD has recently been used to analyze metabolomics measurements collected at multiple time points before and after an oral glucose challenge test from twenty subjects (Fujita et al., 2023). Additional constraints on the factor matrices such as orthogonality as in PCA or HOSVD imposed for uniqueness are not necessarily realistic assumptions, often limiting the interpretability of the methods, especially when the goal is to interpret one component at a time rather than to find subspaces.

The *model fit* shows how well the CP model describes the data and is defined as follows:

$$\begin{aligned} \text {Fit(\%)}=(1-\frac{\left\Vert \, {\varvec{\mathscr{X}}}-\sum _{r=1}^{R} {\varvec{\mathbf {\MakeLowercase {a}}}}_{r}\circ {\varvec{\mathbf {\MakeLowercase {b}}}}_{r} \circ {\varvec{\mathbf {\MakeLowercase {c}}}}_{r} \, \right\Vert ^2}{\left\Vert \, {\varvec{\mathscr{X}}} \, \right\Vert ^2})\times 100, \end{aligned}$$

where $\Vert \cdot \Vert$ denotes the Frobenius norm. If the model fully explains the data, the fit is $100\%$; otherwise, the unexplained part remains in the residuals.

When reporting the results, we normalize each vector, ${\varvec{\mathbf {\MakeLowercase {A}}}}_{r}$, ${\varvec{\mathbf {\MakeLowercase {B}}}}_{r}$, ${\varvec{\mathbf {\MakeLowercase {C}}}}_{r}$, by its norm due to the scaling ambiguity.

#### Model selection

Choosing the right number of components, i.e., *R*, is crucial to extract the underlying patterns accurately; however, the selection of *R* in a CP model is a challenging task (Håstad, 1990; Kolda & Bader, 2009). Here, we choose *R* based on the *replicability* of the extracted components, where *replicability* is defined as the ability to extract similar patterns from random subsamples of the dataset (Adali et al., 2022), and is an extension of split-half analysis (Harshman & De Sarbo, 1984).

The replicability of an *R*-component CP model is assessed as follows:

Step 1.: Randomly split all subjects into ten parts (if there is a class information of potential interest, randomly split the subjects such that each part has the same class proportions as in the original data)
Step 2.: Leave out one part at a time, and form a subset using the remaining data, i.e., in total, ten subsets,
Step 3.: Fit an *R*-component CP model to each subset,
Step 4.: Calculate the similarity of factors between every pair of CP models (in the *metabolites* and *time points* modes), i.e., 45 similarity scores,
Step 5.: Repeat Step 1–4 ten times using different random splitting of subjects in Step 1.

The similarity of the factors in *metabolites* and *time points* modes from two CP models $\llbracket {\varvec{\mathbf {\MakeUppercase {A}}}}, {\varvec{\mathbf {\MakeUppercase {B}}}}, {\varvec{\mathbf {\MakeUppercase {C}}}} \rrbracket$ and $\llbracket \bar{{\varvec{\mathbf {\MakeUppercase {A}}}}}, \bar{{\varvec{\mathbf {\MakeUppercase {B}}}}}, \bar{{\varvec{\mathbf {\MakeUppercase {C}}}}} \rrbracket$ is measured using the factor match score (FMS) after finding the best matching permutation:

$${\text{FMS}} = \frac{1}{R}\sum\limits_{{r = 1}}^{R} {\frac{{\left| {\mathbf{b}_{r} ^{T} \mathbf{\bar{b}}_{r} } \right|}}{{\left\| {\mathbf{b}_{r} } \right\|\left\| {\mathbf{\bar{b}}_{r} } \right\|}}} \frac{{\left| {\mathbf{c}_{r} ^{T} \mathbf{\bar{c}}_{r} } \right|}}{{\left\| {\mathbf{c}_{r} } \right\|\left\| {\mathbf{\bar{c}}_{r} } \right\|}}.$$

An *R*-component CP model is considered to be replicable if $95\%$ of FMS values (of 450 FMS values computed at the end of Step 5) is higher than 0.9. We choose the highest number of components that produces a replicable CP model in order to explain the data as much as possible. There are various diagnostic approaches used to determine the number of CP components such as the core consistency diagnostic (Bro & Kiers, 2003), and the increase in model fit. These approaches are often used together since no single method can always give the right number of components. Here, we primarily rely on a replicability-based model selection approach since the replicability of the extracted patterns is particularly important when the goal is to reveal biomarkers.

### Experimental setting

Before the analysis, the data is preprocessed by first centering across the *subjects* mode and then scaling within the *metabolites* mode (Bro & Smilde, 2003). When scaling, each slice in the *metabolites* mode is divided by the root mean squared value of non-missing entries.

All experiments are performed in MATLAB (2020b). For fitting the CP model, we use cp-wopt (Acar et al., 2011) from the Tensor Toolbox (version 3.1) (Bader & Kolda, 2022) using the nonlinear conjugate gradient algorithm from the Poblano Toolbox (Dunlavy et al., 2010). Multiple random initializations are used when fitting the model to avoid local minima, and the run returning the minimum function value is used for further analysis. See the Github repository^3^ for details. For PCA, we use the MATLAB function svd after estimating the missing values using weighted optimization (Acar et al., 2011).

## Results

### The CP model of T0-corrected metabolomics data from males reveals a BMI-related group difference

We analyze the T0-corrected data from males using a 2-component CP model. The number of components is selected based on the replicability of extracted patterns. See Supplementary Material S2 for more details on model selection. The model fit is 44%.

**Fig. 2:**
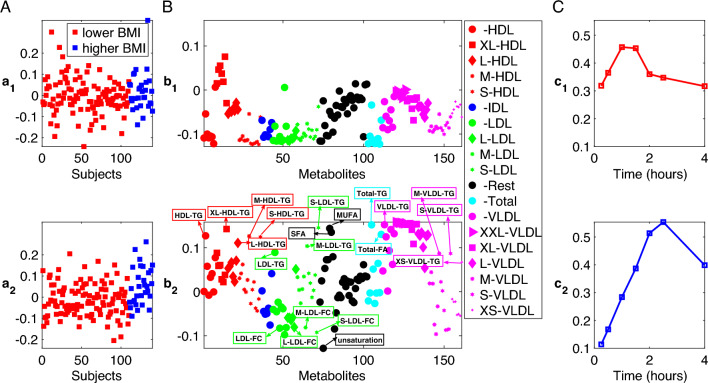
2-component CP model of the T0-corrected metabolomics data from males. **A** *Subjects* mode (i.e., ${\varvec{\mathbf {\MakeLowercase {a}}}}_{1}$ and ${\varvec{\mathbf {\MakeLowercase {a}}}}_{2}$), where subjects are colored according to BMI defined as lower BMI: BMI$<25$ and higher BMI: BMI $\ge 25$, **B** *Metabolites* mode (i.e., ${\varvec{\mathbf {\MakeLowercase {b}}}}_{1}$ and ${\varvec{\mathbf {\MakeLowercase {b}}}}_{2}$), where metabolites are colored according to lipoprotein classes. The size of the marker for each metabolite is adjusted according to lipoprotein subclasses as indicated in the legend. We show the names of the metabolites with the highest coefficients for the component of interest, i.e., ${\varvec{\mathbf {\MakeLowercase {b}}}}_{2}$. The Rest group contains features other than the ones in the lipoprotein group, the Total group corresponds to total concentrations of certain metabolites, e.g., Total-C, Total-TG. See Tabel S1.1 in Supplementary Material S1 for details, **C** *Time* mode (i.e., ${\varvec{\mathbf {\MakeLowercase {c}}}}_{1}$ and ${\varvec{\mathbf {\MakeLowercase {c}}}}_{2}$)

**Fig. 3:**
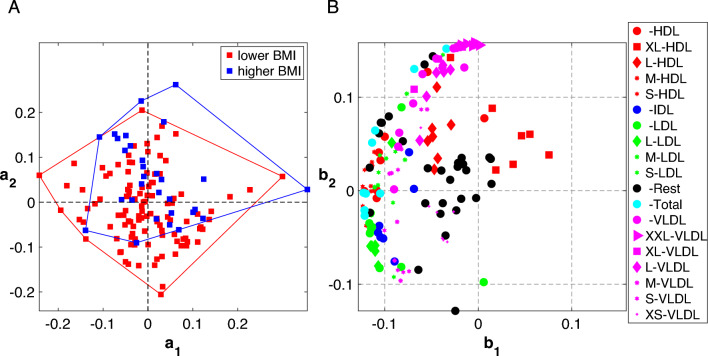
Scatter plots of the first and second component from the 2-component CP model of the T0-corrected data from males. **A** *Subjects* mode, (i.e., ${\varvec{\mathbf {\MakeLowercase {a}}}}_{1}$ and ${\varvec{\mathbf {\MakeLowercase {a}}}}_{2}$) colored according to BMI defined as lower BMI: BMI$<25$ and higher BMI: BMI $\ge 25$, and **B** *Metabolites* mode, where metabolites are colored according to lipoprotein classes. The size of the marker for each metabolite is adjusted according to lipoprotein subclasses as indicated in the legend. The Rest group contains features other than the ones in the lipoprotein group, the Total group corresponds to total concentrations of certain metabolites, e.g., Total-C, Total-TG. See Table S1.1 in Supplementary Material S1 for details

Figure 2 shows the factors $({\varvec{\mathbf {\MakeLowercase {a}}}}_{r},{\varvec{\mathbf {\MakeLowercase {b}}}}_{r},{\varvec{\mathbf {\MakeLowercase {c}}}}_{r})$ extracted by the 2-component CP model. To see whether the model reveals any underlying group structure among subjects, we consider additional information available about the participants after the analysis. In the *subjects* mode (i.e., ${\varvec{\mathbf {\MakeLowercase {a}}}}_{1}$ and ${\varvec{\mathbf {\MakeLowercase {a}}}}_{2}$) in Fig. 2, subjects are colored according to BMI groups, i.e., *lower* vs. *higher* BMI groups. The lower BMI group contains subjects with BMI less than 25.0 (i.e., underweight and normal weight subjects) while the higher BMI group contains subjects with BMI greater than or equal to 25.0 (i.e., overweight and obese subjects). In the *metabolites* mode (i.e., ${\varvec{\mathbf {\MakeLowercase {b}}}}_{1}$ and ${\varvec{\mathbf {\MakeLowercase {b}}}}_{2}$), metabolites are colored according to lipoprotein subclasses. Here, the **Rest** group contains features other than the ones in the lipoprotein group while the **Total** group consists of total concentrations of certain metabolites, e.g., Total-C, Total-TG. See Supplementary Material S1 (Table S1.1) for details. In the *time* mode (i.e., ${\varvec{\mathbf {\MakeLowercase {c}}}}_{1}$ and ${\varvec{\mathbf {\MakeLowercase {c}}}}_{2}$), the model reveals the temporal patterns.

In this model, the second CP component, i.e., (${\varvec{\mathbf {\MakeLowercase {A}}}}_{2}$, ${\varvec{\mathbf {\MakeLowercase {B}}}}_{2}$, ${\varvec{\mathbf {\MakeLowercase {C}}}}_{2}$), is of particular interest since it reveals a statistically significant group difference (*p*-value $=6 \times 10^{-4}$ using a two-sample *t*-test based on ${\varvec{\mathbf {\MakeLowercase {A}}}}_{2}$) in terms of BMI. Supplementary Material S3—Fig. S3.4 gives a more detailed visualization of the second component. In the *metabolites* mode, i.e., ${\varvec{\mathbf {\MakeLowercase {B}}}}_{2}$ in Fig. 2B, we observe that the subject group difference is due to different behaviour of certain metabolites, i.e., metabolites with high coefficients in terms of absolute value. In particular, we observe that TG-related metabolites, VLDLs (XXL/XL/L), monounsaturated fatty acids (MUFA), saturated fatty acids (SFA), and total fatty acids (Total-FA) have high positive coefficients (indicating that changes in these metabolites positively relate to higher BMI) while several LDL-FCs, VLDLs (M/S), and unsaturation degree have high negative coefficients (indicating that changes in these metabolites negatively relate to higher BMI). Unsaturation degree is the level of unsaturation of fatty acids within a sample, high levels of which indicating that the fatty acid content is likely to be polyunsaturated rather than saturated for a specific sample. Time profiles of the raw data for these specific metabolites, i.e., the ones with high coefficients in terms of absolute value, are included in Supplementary Material S3. In the *time* mode, ${\varvec{\mathbf {\MakeLowercase {c}}}}_{2}$ increases gradually until 2.5 h and decreases afterwards showing the temporal profile of the metabolic response modelled by this component.

Note that while we discuss subject group differences in terms of BMI, this component also has significant correlation with several other variables. Figure 6 shows the correlation of the factor vector in the *subjects* mode for the second component, i.e., ${\varvec{\mathbf {\MakeLowercase {A}}}}_{2}$, with various variables of interest. High positive and negative correlations indicate that the second component is related to not only BMI but also a phenotype defined by these closely related variables.

The first component, i.e., (${\varvec{\mathbf {\MakeLowercase {A}}}}_{1}$, ${\varvec{\mathbf {\MakeLowercase {B}}}}_{1}$, ${\varvec{\mathbf {\MakeLowercase {C}}}}_{1}$), on the other hand, models an earlier response captured by ${\varvec{\mathbf {\MakeLowercase {c}}}}_{1}$ potentially modelling individual differences. Scatter plots of the first and second component in the *subjects* and *metabolites* modes showing subject groups and groups of lipoproteins based on their classes and subclasses are given in Fig. 3.

### Analysis of the fasting state metabolomics data from males using PCA reveals a BMI-related group difference

**Fig. 4:**
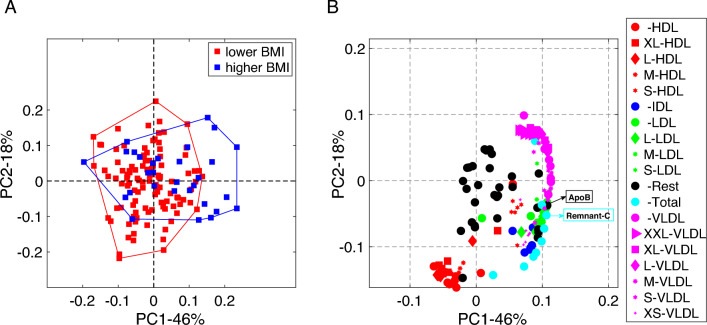
Scatter plots from PCA of the fasting state data from males: **A** *Subjects* mode, where subjects are colored according to BMI defined as lower BMI: BMI$<25$ and higher BMI: BMI $\ge 25$, and **B** *Metabolites* mode, where metabolites are colored according to lipoprotein classes. The size of the marker for each metabolite is adjusted according to lipoprotein subclasses as indicated in the legend. We show the names of the metabolites with the highest coefficients for the first component, where we observe a statistically significant group difference in terms of BMI. The Rest group contains features other than the ones in the lipoprotein group, and the Total group corresponds to total concentrations of certain metabolites, e.g., Total-C, Total-TG. See Supplementary Material S1 (Table S1.1) for details

Figure 4 shows the scatter plots of principal components in the *subjects* and *metabolites* modes from the PCA of the fasting state data. In the *subjects* mode, a weak but statistically significant (*p*-value $=1\times 10^{-3}$) group difference is captured using the first principal component (PC1). In the *metabolites* mode, we observe that in PC1, IDLs, LDLs, VLDLs, Apolipoprotein B (ApoB), and Remnant cholesterol (Remnant-C) have high positive loadings showing positive relation with the concentrations of these metabolites and the higher BMI group.

**Fig. 5:**
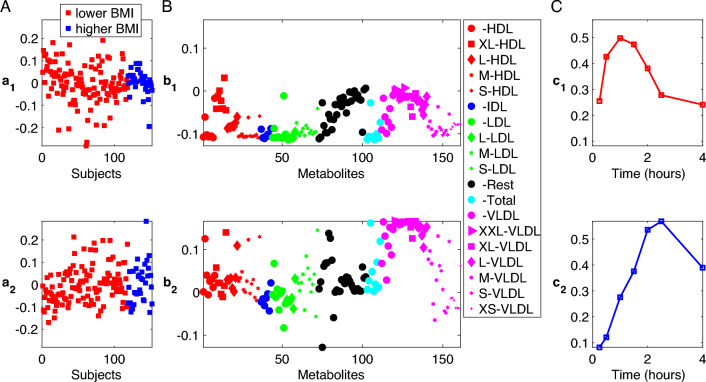
2-component CP model of the T0-corrected metabolomics data from females. **A** *Subjects* mode (i.e., ${\varvec{\mathbf {\MakeLowercase {a}}}}_{1}$ and ${\varvec{\mathbf {\MakeLowercase {a}}}}_{2}$), where subjects are colored according to BMI defined as lower BMI: BMI$<25$ and higher BMI: BMI $\ge 25$, **B** *Metabolites* mode (i.e., ${\varvec{\mathbf {\MakeLowercase {b}}}}_{1}$ and ${\varvec{\mathbf {\MakeLowercase {b}}}}_{2}$), where metabolites are colored according to lipoprotein classes. The size of the marker for each metabolite is adjusted according to lipoprotein subclasses as indicated in the legend. The Rest group contains features other than the ones in the lipoprotein group, the Total group corresponds to total concentrations of certain metabolites, e.g., Total-C, Total-TG, and **C** *Time* mode (i.e., ${\varvec{\mathbf {\MakeLowercase {c}}}}_{1}$ and ${\varvec{\mathbf {\MakeLowercase {c}}}}_{2}$)

### The CP model of T0-corrected metabolomics data from females does not reveal a BMI-related group difference

Factors captured by the analysis of the T0-corrected data from females using a 2-component CP model are given in Fig. 5 (see Supplementary Material S2 for the selection of the number of components). The fit of the 2-component model is $47\%$.

**Fig. 6:**
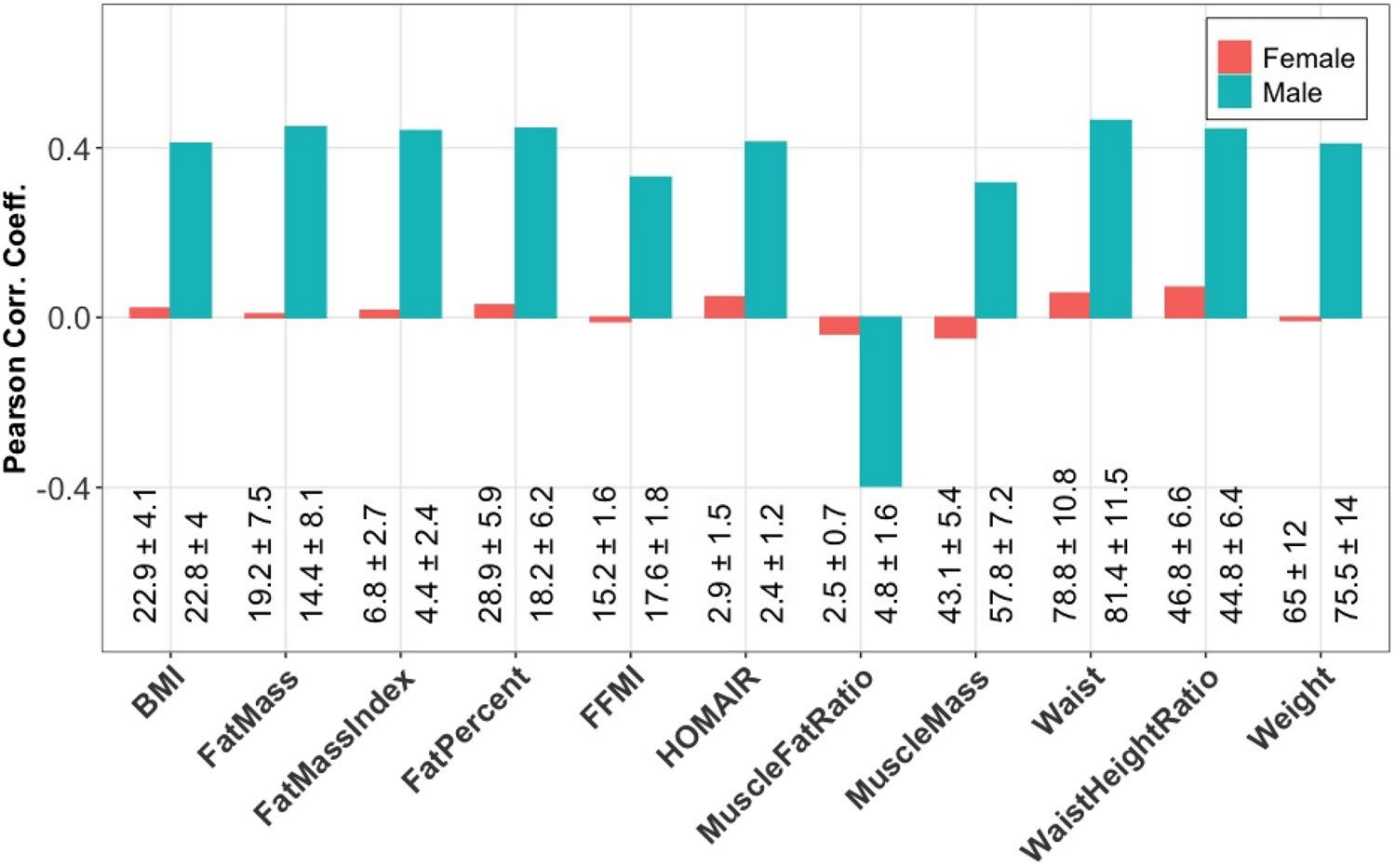
Correlation between the second component (${\varvec{\mathbf {\MakeLowercase {a}}}}_{2}$) of the 2-component CP model of the T0-corrected data in the *subjects* mode with the additional meta data. Measurements from males and females are analyzed using a CP model separately as shown in Figs. 2 and 5. The second component vector in the *subjects* mode, i.e., (${\varvec{\mathbf {\MakeLowercase {a}}}}_{2}$), from each model is used to compute the correlations for males and females. Descriptions of these variables are as follows: *HOMAIR* homeostatic model assessment for Insulin Resistance; *MuscleFatRatio* muscle to fat ratio; *FatPercent* body fat percentage; *MuscleMass* amount of muscle in the body (kg); Weight (kg); *BMI* Body Mass Index; *Waist* Waist circumferance (cm); *WaistHeightRatio* waist measurement divided by height (cm); *FatMass* amount of body fat (kg); *FatMassIndex; FFMI* Fat Free Mass Index. The mean ± standard deviation for each variable and sex is shown at the bottom of the plot, and more details are provided in Table S1.2 in Supplementary Material S1

In the *subjects* mode, we do not observe a group difference in either component in terms of the additional information available about the participants, see ${\varvec{\mathbf {\MakeLowercase {a}}}}_{1}$ and ${\varvec{\mathbf {\MakeLowercase {a}}}}_{2}$ in Fig. 5A, where subjects are colored according to *lower* and *higher* BMI groups. Note that correlations are also quite low for females in Fig. 6. The *time* mode shows two underlying profiles: early response in ${\varvec{\mathbf {\MakeLowercase {c}}}}_{1}$, and a later response in ${\varvec{\mathbf {\MakeLowercase {c}}}}_{2}$. Even though the second component is similar in the *metabolites* and *time* modes, i.e., ${\varvec{\mathbf {\MakeLowercase {B}}}}_{2}$ and ${\varvec{\mathbf {\MakeLowercase {C}}}}_{2}$ in Fig. 5, to the component captured by the CP model of the T0-corrected data from males, i.e., (${\varvec{\mathbf {\MakeLowercase {A}}}}_{2}$, ${\varvec{\mathbf {\MakeLowercase {B}}}}_{2}$, ${\varvec{\mathbf {\MakeLowercase {C}}}}_{2}$) in Fig. 2, no BMI-related group difference is observed in this component for females (see Fig. 8 for a clear comparison of the components from males vs. females).

### Analysis of the fasting state metabolomics data from females using PCA reveals a BMI-related group difference

**Fig. 7:**
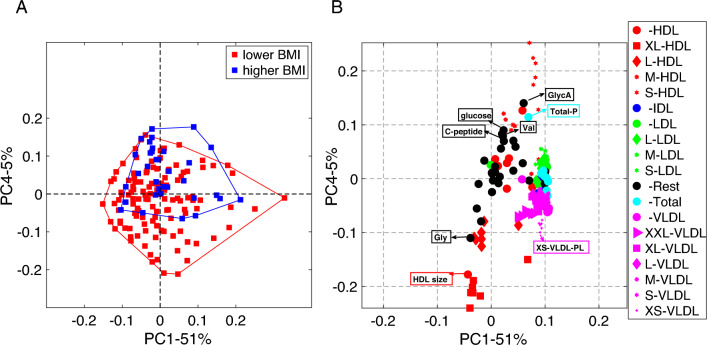
Scatter plots from PCA of the fasting-state data from females: **A** *Subjects* mode, where subjects are colored according to BMI defined as lower BMI: BMI$<25$ and higher BMI: BMI $\ge 25$, and **B** *Metabolites* mode, where metabolites are colored according to lipoprotein classes. The size of the marker for each metabolite is adjusted according to lipoprotein subclasses as indicated in the legend. We show the names of the metabolites with the highest coefficients for the fourth component, where we observe a statistically significant group difference in terms of BMI. The Rest group contains features other than the ones in the lipoprotein group, the Total group corresponds to total concentrations of certain metabolites, e.g., Total-C, Total-TG

Scatter plots of PC1 and PC4 in the *subjects* and *metabolites* modes from PCA of the fasting state data from females are shown in Fig. 7. In the *subjects* mode, only PC4 reveals a statistically significant (*p*-value $=5\times 10^{-6}$) BMI-related group difference. Note that the fourth component only explains 5% of the variation in the data so the signal—although statistically significant—is weak in this data set. In the *metabolites* mode, we observe that in PC4, HDLs (S), C-peptide, glucose, glycoprotein acetyls (GlycA), total concentration of lipoprotein particles (Total-P), and valine (Val) have high positive loadings (positively relate to *higher* BMI) while the HDLs (XL), average diameter for HDL particles (HDL size), glycine (Gly), and XS-VLDL-PL have high negative loadings (negatively relate to *higher* BMI).

We summarize the metabolites observed to be related to BMI-related group difference in Table 1.

**Table 1:** Metabolites with high coefficients (in terms of absolute value) observed to be related to BMI-related group difference

	T0-corrected		Fasting state	
Males	(+)	TG-related, Total-FA, MUFA	(+)	IDLs, LDLs, VLDLs
		SFA,VLDLs (XXL/XL/L)		ApoB, Remnant-C
	(−)	LDL-FC-related, VLDLs (M/S)		
		unsaturation		
Females			(+)	HDLs (S), GlycA, glucose
				C-peptide, Total-P, Val
			(−)	HDLs (XL), HDL size
				Gly, XS-VLDL-PL

(+) and (−) denotes positive and negative relation to the higher BMI group, respectively

## Discussion

### Fasting vs. dynamic states

In males, groups of lipoproteins reveal BMI-related group difference, and the relations of those lipoproteins with BMI groups differ in the fasting vs. dynamic state. For the fasting state data, the positive relation of LDL-C (Bays et al., 2013; Lamon-Fava et al., 1996), VLDL-C (Lamon-Fava et al., 1996) and ApoB (Lamon-Fava et al., 1996) with higher BMI has been previously reported, and our findings are consistent with the literature. Likewise, we find similar patterns whereby large triglyceride rich VLDL lipoproteins are increased in individuals with higher BMI following a meal challenge containing fats assessed via conventional AUC postprandial metrics (Wang et al., 2017). However, we did not find complementary decreases in HDL, this may be in part due to gender stratification differences in our analyses. Likewise monozygotic twin studies point to that these observed differences in lipoprotein responses are driven by BMI differences, rather than underlying genetics (Rämö et al., 2017).

While Table 1 gives a summary of the most important metabolites, i.e. metabolites with high coefficients in terms of absolute value, we also observe in Fig. 4 that HDLs (XL/L) negatively relate to higher BMI in the fasting state (even though they are not among the most important metabolites; therefore, not reported in Table 1). This matches with previous results in terms of HDL-C (Bays et al., 2013; Lamon-Fava et al., 1996).

This study differs from previous challenge test studies as it is a comprehensive study in terms of the lipoproteins (classes and subclasses) considered in the analysis, and the comprehensive picture provided by the CP model from the postprandial data as well as its comparison with the fasting state analysis. We observe that dynamic and fasting state data provide different views of the same set of metabolites. These different views reveal different relations with the BMI groups as we observe in the case of VLDLs, i.e., while concentrations of all VLDLs positively relate to higher BMI in the fasting state (see Fig. 4), Fig. 2 shows that changes in VLDLs (XXL/XL/L) positively relate to higher BMI, and changes in VLDLs (M/S) negatively relate to higher BMI in the dynamic state.

These results not only improve our understanding of dynamic changes in the human metabolome but also reveal dynamic and static markers through the analysis of fasting and postprandial data.

### Males vs. females (dynamic state)

**Fig. 8:**
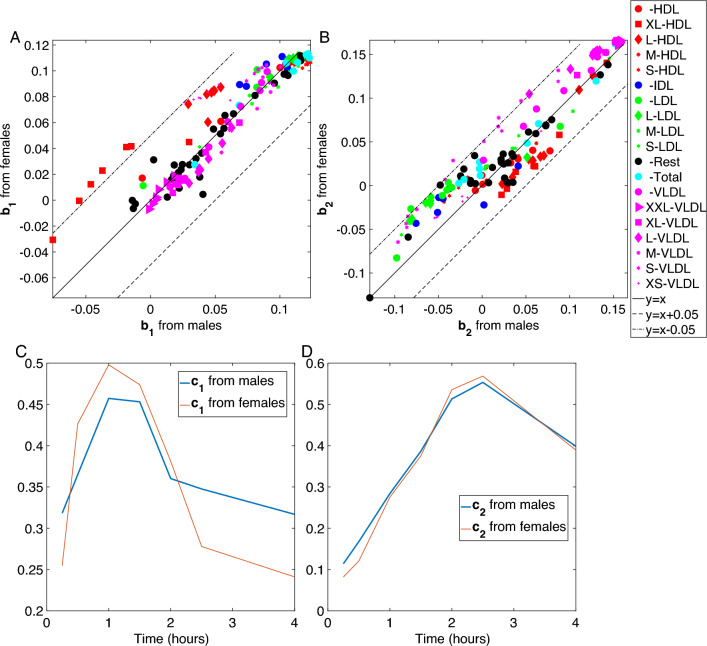
Comparison of CP models of T0-corrected data from males and females. **A** and **B** *Metabolites* mode. For better comparison, the line $y=x$ is plotted together with $y=x-0.05$ and $y=x+0.05$. **C** and **D** *Time* mode

Patterns of dynamic response to the challenge test, i.e., patterns in the *metabolites* and *time* modes in T0-corrected analyses, are similar in males vs. females. Figure 8 compares the components extracted using CP models from males vs. females in the *metabolites* and *time* modes. Figure 8A and C show the comparison of ${\varvec{\mathbf {\MakeLowercase {b}}}}_{1}$ from males vs. females, and ${\varvec{\mathbf {\MakeLowercase {c}}}}_{1}$ from males vs. females, respectively. The second components (i.e., ${\varvec{\mathbf {\MakeLowercase {b}}}}_{2}$ and ${\varvec{\mathbf {\MakeLowercase {c}}}}_{2}$), which are the patterns of interest in terms of BMI-related group difference, are compared in Fig. 8B and D. We observe similar patterns in both modes indicating that patterns of dynamic response to the challenge test are similar in males and females.

We strengthen this argument by analyzing the T0-corrected data from all individuals (both males and females) using a CP model. When males and females are analyzed together, the components in the *metabolites* and *time* modes are the same for both sexes. In the *subjects* mode, BMI-related group difference is observed among males but still not among females (See Supplementary Material S4).

The fat distribution is usually quite different for males and females, with overweight males often having central obesity, i.e., adiposity (consisting of TGs) in and around the liver, where VLDL is produced. Young females have more subcutaneous fat than men and also deposit more fat non-centrally, e.g., on other places such as hips, thighs etc.; pre-menopausal women also export less TG from the liver, because they deposit it elsewhere, and the VLDL may therefore contain less TG and pack more efficiently, so the observed gender differences can be linked to the effect of such anthropometric differences on TG metabolism (Palmisano et al., 2018). The presence of gender differences can also be anticipated due to the inherent variances in sex hormones, psychosocial factors, and cardiovascular risk factor profiles (Loh et al., 2022). These postprandial gender differences may in part explain the cardiovascular risk reduction noted in pre-menopausal women (Zaman et al., 2012). Note that our cohort comprises individuals who are 18 years old from both sexes, and this uniformity allows us to infer that the observed sex-based differences might mirror the variations in cardiovascular risk profiles. While our study’s strength lies in the uniform age of the cohort, further research is needed to clarify these relationships and to better understand how our findings might extend to different age demographics and clinical domains.

### Males vs. females (fasting state)

In the fasting state, the main patterns in the *metabolites* mode are similar in males vs. females while the relations of those patterns with BMI differ. Figures 4 and 7 show the scatter plots of PCs in the fasting state analysis of males and females, respectively. Similar patterns are observed for both males and females. More precisely, in PC1s (in Figs. 4 and 7), IDLs, LDLs, and VLDLs are the metabolites with high positive coefficients. While PC1 shows strong signals from lipoproteins in both males and females, it shows BMI-related group difference only in males. This could again be linked to anthropometric differences between genders. BMI in overweight young women is not strongly associated with abdominal obesity so we do not see PC1 as related to BMI. In men, abdominal fat is strongly associated with BMI, so BMI is a good proxy. This component is not only related to BMI but also to waist circumference, fat mass, and fat mass index which would all indicate that excess adiposity is probably more relevant to this component than BMI (which is also affected by muscle mass).

In females, PC4 in Fig. 7 reveals a statistically significant group difference in terms of BMI, and the metabolites with high positive coefficients are mainly the HDLs (S) and the ones with high negative coefficients are mainly the HDLs (XL). For males, we also observe in PC1 (even though the coefficients are small) that HDLs (S) positively relate to higher BMI while HDLs (XL) negatively relate to the higher BMI group capturing the relation revealed by PC4 in females. Thus, for both males (from PC1 in Fig. 4) and females (from PC4 in Fig. 7), we can say that HDLs (S) are positively related to higher BMI and HDLs (XL) are negatively related to higher BMI. In addition, in PC4 for females, we observe metabolites related to insulin sensitivity and inflammation, indicating that the obese and overweight women have higher glucose, C-peptide (marker of insulin synthesis), valine (a branched-chain amino acid known to increase in subjects being less insulin-sensitive (Zhao et al., 2016)), and GlycA (an inflammatory marker (Otvos et al., 2015))

These differences between genders may also be explained in the pre-menopausal cardioprotective lipid profiles of females (Kilim & Chandala, 2013). This gender disparity in postprandial atherogenic remnant lipoproteins may help explain why males are at greater cardiovascular risk in early life. Literature suggests gender differences in cardiovascular risk are abolished after menopause (Rich-Edwards et al., 1995), and this may point towards the sex hormones playing a moderating role on atherogenic lipoproteins.

## Conclusion

In this study, we have demonstrated a comprehensive analysis of metabolomics measurements collected during a challenge test from the COPSAC_2000_ cohort. We have arranged time-resolved metabolomics measurements as a *subjects* by *metabolites* by *time points* tensor, and analyzed fasting state-corrected dynamic data using a CP model in comparison with the fasting state data. Our analysis demonstrates that the CP model reveals interpretable patterns showing how subject groups (i.e., lower vs. higher BMI groups) differ in terms of the dynamic response of certain metabolite groups. We also show that metabolites behave differently in fasting vs. dynamic states; therefore, analysis of fasting and postprandial data can reveal static as well as dynamic biomarkers. In addition, our results indicate that patterns of dynamic response to the challenge test are similar in males vs. females but differences are observed in terms of how those patterns relate to BMI groups. Extracted patterns have shown to be not only interpretable but also replicable.

While the CP model can reveal a comprehensive picture of time-resolved postprandial data and novel insights such as sex differences and metabolite groups behaving differently in fasting vs. dynamic states, there are still several limitations. The set of measured metabolites/features is dominated by lipoproteins; therefore, the main patterns of variation in the data mainly model the behavior of lipoproteins, which is the case for both CP and PCA. We do not observe glycolysis-related metabolites, amino acids or ketone bodies well-modelled due to the dominant set of lipoproteins. In order to get a better understanding of postprandial metabolic response in terms of glycolysis, ketogenesis, and the aminoacid metabolism, metabolites may be grouped into specific subsets and jointly analyzed. Also, in this study, we have mainly focused on one pattern,i.e., one CP component or PCA component, at a time (even if scatter plots are considered). However, multiple components together may explain the meta variables of interest, e.g., BMI groups, better. For both males and females, when multiple linear regression is used to predict BMI values using the factor matrix in the *subjects* mode, we do not observe any improvement using multiple components. However, in PCA, multiple components can be relevant. As we are not interested in a specific variable but rather in capturing components that may relate to a certain phenotype (characterized by multiple variables), we have focused on one component at a time and studied the correlation of the subject scores with the available meta variables of interest here. Depending on the goal, multiple components can be clustered or used in further prediction tasks. We should also mention that as an unsupervised method, the CP model focuses on the main patterns explaining the variation in the data. These main patterns may not necessarily be related to meta variables of interest. Such an unsupervised approach may miss the relation with meta variables if that relation does not significantly contribute to the main variation in the data. In those cases, supervised methods may perform better. However, the goal of using an unsupervised approach in this exploratory study is its potential to reveal unknown subject stratifications rather than predicting meta variables.

Another future research direction to consider is the joint analysis of postprandial metabolomics data with other omics data sets, in particular, gut microbiome data, through data fusion methods (Acar et al., 2015; Schenker et al., 2021). With recent technological advances, e.g., multi-omics microsampling (Shen et al., 2023), that may make such challenge test data more easily available, jointly analyzing dynamic metabolomics data with other omics data sets becomes even more crucial.

## Supplementary information

Supplementary files contain the full list of metabolites, descriptive statistics of meta variables, selection of the number of components for the CP model, supplementary figures and comparison of CP models from males and females.

### Supplementary Information

Below is the link to the electronic supplementary material.

Supplementary file 1 (xlsx 14 KB)

Supplementary file 2 (pdf 458 KB)

Supplementary file 3 (pdf 2415 KB)

Supplementary file 4 (pdf 174 KB)

## Data Availability

NMR measurements of plasma samples are not publicly available but may be shared by COPSAC through a collaboration agreement. Data access requests should be directed to Morten A. Rasmussen (morten.arendt@dbac.dk). The GitHub repository https://github.com/eacarat/CP_Metabolomics_MealChallenge contains the scripts showing the preprocessing steps and analyzing the data using a CP model as well as the scripts for replicability.

## Footnotes

1 While our study is nested within the COPSAC_2000_ mother-child cohort, which at its inception recruited mothers with a history of asthma, this current study is not intended, nor describes any connections with asthma.

2 The full list of metabolites is available at https://research.nightingalehealth.com/biomarkers.

3 https://github.com/eacarat/CP_Metabolomics_MealChallenge.
