# Supplementary material for: Characterizing human postprandial metabolic response using multiway data analysis
Source: Metabolomics. 2024 May 9;20(3):50. doi: 10.1007/s11306-024-02109-y (PMC11082008; doi:10.1007/s11306-024-02109-y)
Supplement: Supplementary file 2 — (pdf 458 KB) [file 11306_2024_2109_MOESM2_ESM.pdf]

Supplementary Material S2:
Component Number Selection in CP Models

1 CP model of the T0-corrected metabolomics data from males

When selecting the number of components, we rely on the replicability of the patterns extracted using the CP model. Figure S2.1a shows the replicability of the CP model of T0-corrected data from males for different number of components. Models are replicable for $R = 1$ and $R = 2$. We choose the replicable model with the highest number of components (i.e., $R = 2$) as the 2-component model has a much higher model fit than the one-component model as shown in Figure S2.1b.

One potential issue that may be encountered when fitting a CP model is degeneracy [2]. Typically, in such a case, factors become proportional, i.e., the Tucker's congruence (TC)[1] given in Equation 1 between a pair of components gets close to -1. The TC value between component i and component j is defined as follows:

$$\mathrm{TC}_{ij} = \frac{\mathbf{a}_i{}^{\mathsf{T}}\mathbf{a}_j}{\parallel \mathbf{a}_i \parallel \parallel \mathbf{a}_j \parallel} \frac{\mathbf{b}_i{}^{\mathsf{T}}\mathbf{b}_j}{\parallel \mathbf{b}_i \parallel \parallel \mathbf{b}_j \parallel} \frac{\mathbf{c}_i{}^{\mathsf{T}}\mathbf{c}_j}{\parallel \mathbf{c}_i \parallel \parallel \mathbf{c}_j \parallel}, \tag{1}$$

where $(\mathbf{a}_i,\ \mathbf{b}_i,\ \mathbf{c}_i)$ denotes the ith component of an R-component CP model.

When selecting the number of components, we make sure that the model is not degenerate. For instance, in males, we observe degeneracy with $R = 3$ (with TC value around -0.8) and $R = 4$ (with TC value around -0.99) components. This also supports using $R = 2$ for the number of components.

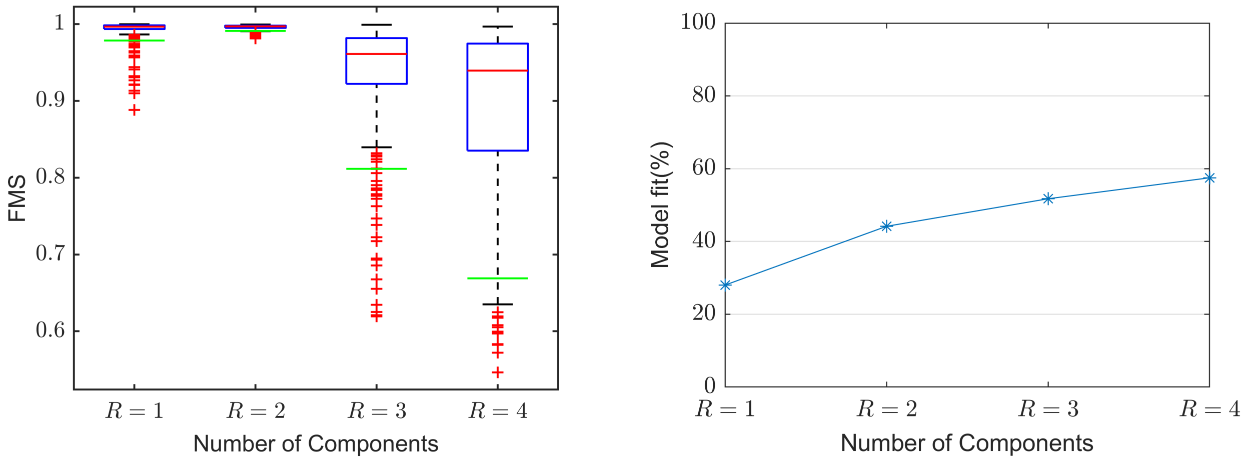

(a) Replicability

(b) Model Fit

Figure S2.1: CP model of T0-corrected data from males. Green lines show that 95% of FMS values are above those lines.

2 CP model of the T0-corrected metabolomics data from females

Figure S2.2a shows the replicability of the CP model of the T0-corrected data from females for different number of components, R. The model is replicable for $R = 1, 2, 4$. The 2-component CP model is chosen here because the 4-component model is degenerate (with a TC value of -0.78).

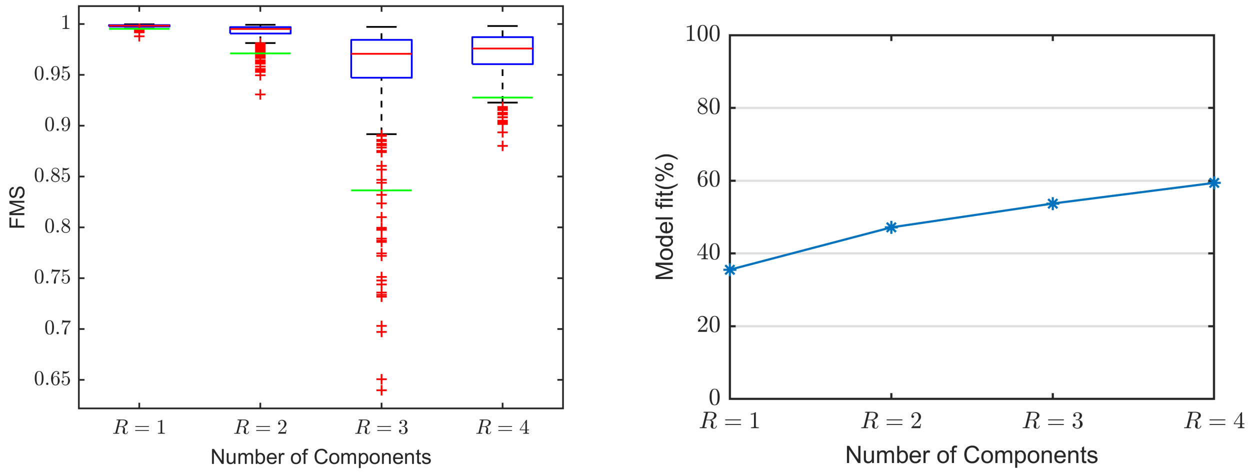

(a) Replicability (b) Model Fit

Figure S2.2: CP model of T0-corrected data from females. Green lines show that 95% of the FMS values are above that line. Models are replicable for $R = 1$, $R = 2$, and $R = 4$

References

[1] R. Bro. *Multi-way analysis in the food industry: models, algorithms, and applications*. PhD thesis, 1998.

[2] T. G. Kolda and B. W. Bader. Tensor decompositions and applications. *SIAM Review*, 51(3):455–500, aug 2009.
