# Supplementary material for: Characterizing human postprandial metabolic response using multiway data analysis
Source: Metabolomics. 2024 May 9;20(3):50. doi: 10.1007/s11306-024-02109-y (PMC11082008; doi:10.1007/s11306-024-02109-y)
Supplement: Supplementary file 3 — (pdf 2415 KB) [file 11306_2024_2109_MOESM3_ESM.pdf]

Supplementary Material S3:
Supplementary Figures

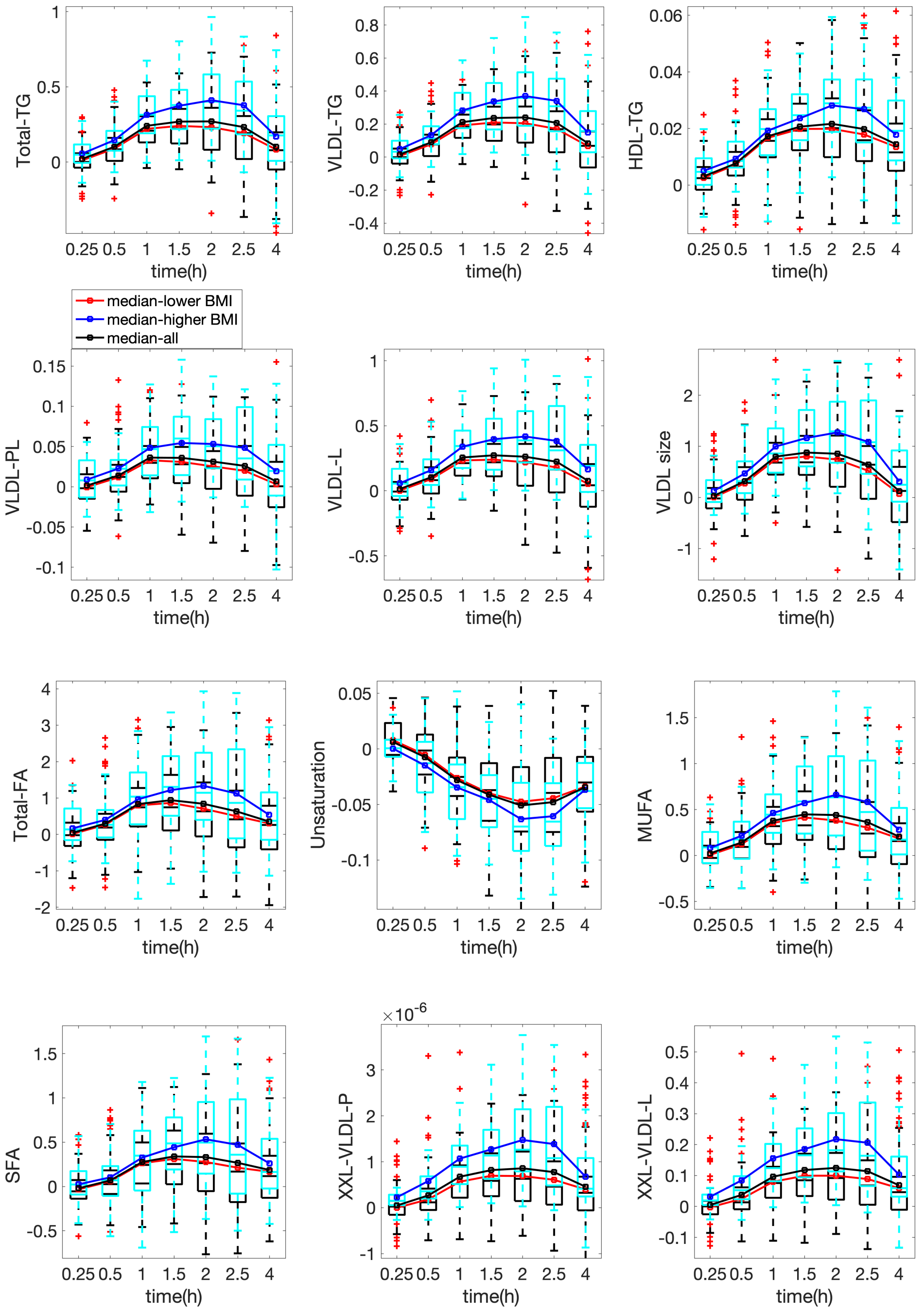

Figure S3.1: Time profiles of selected metabolites (raw data). These belong to the group of metabolites with large absolute coefficients (> 0.1) in $\mathbf{b}_2$ in the 2-component CP model of the T0-corrected data from males.

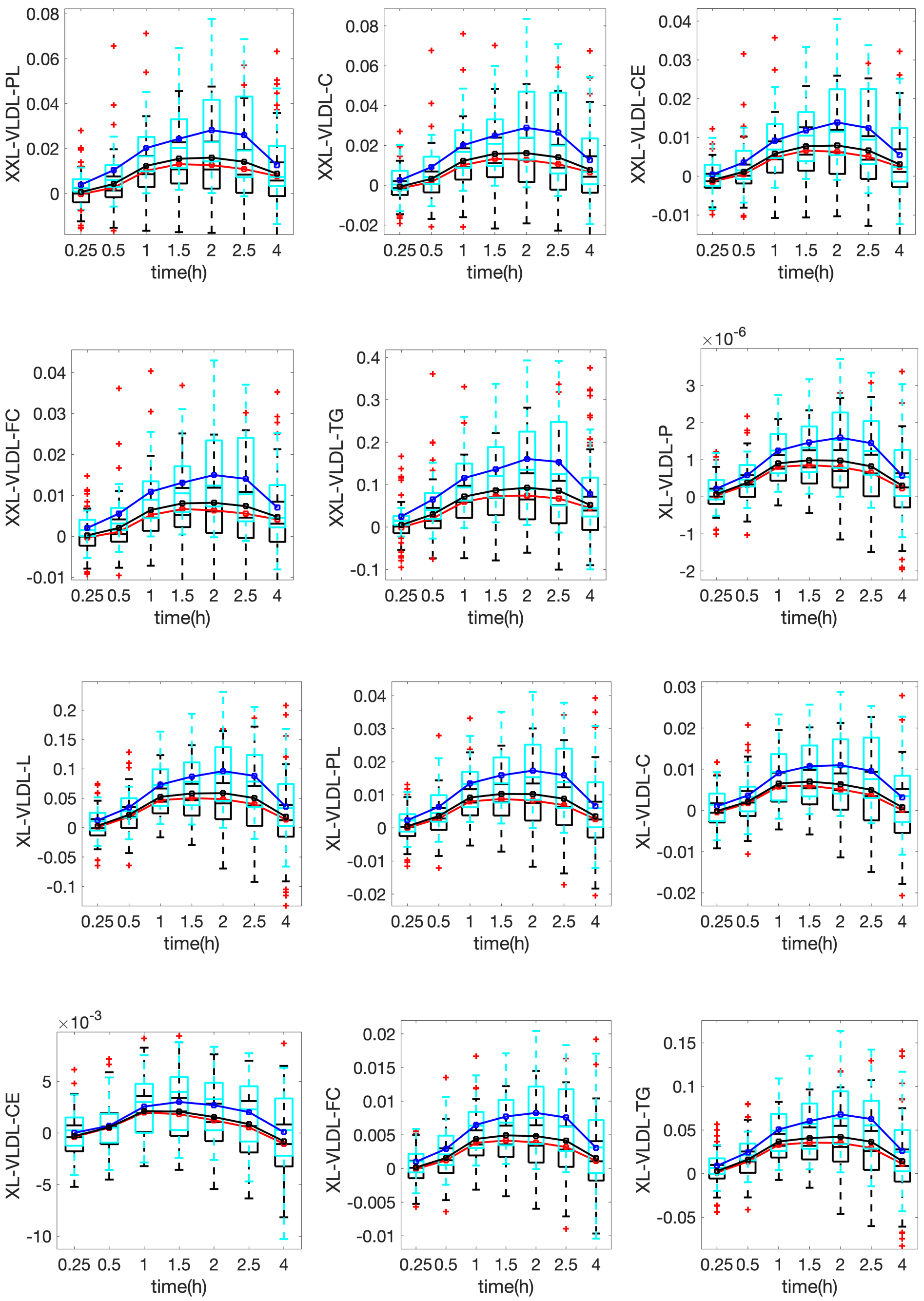

Figure S3.2: Time profiles of selected metabolites (raw data). These belong to the group of metabolites with large absolute coefficients (> 0.1) in $\mathbf{b}_2$ in the 2-component CP model of the T0-corrected data from males.

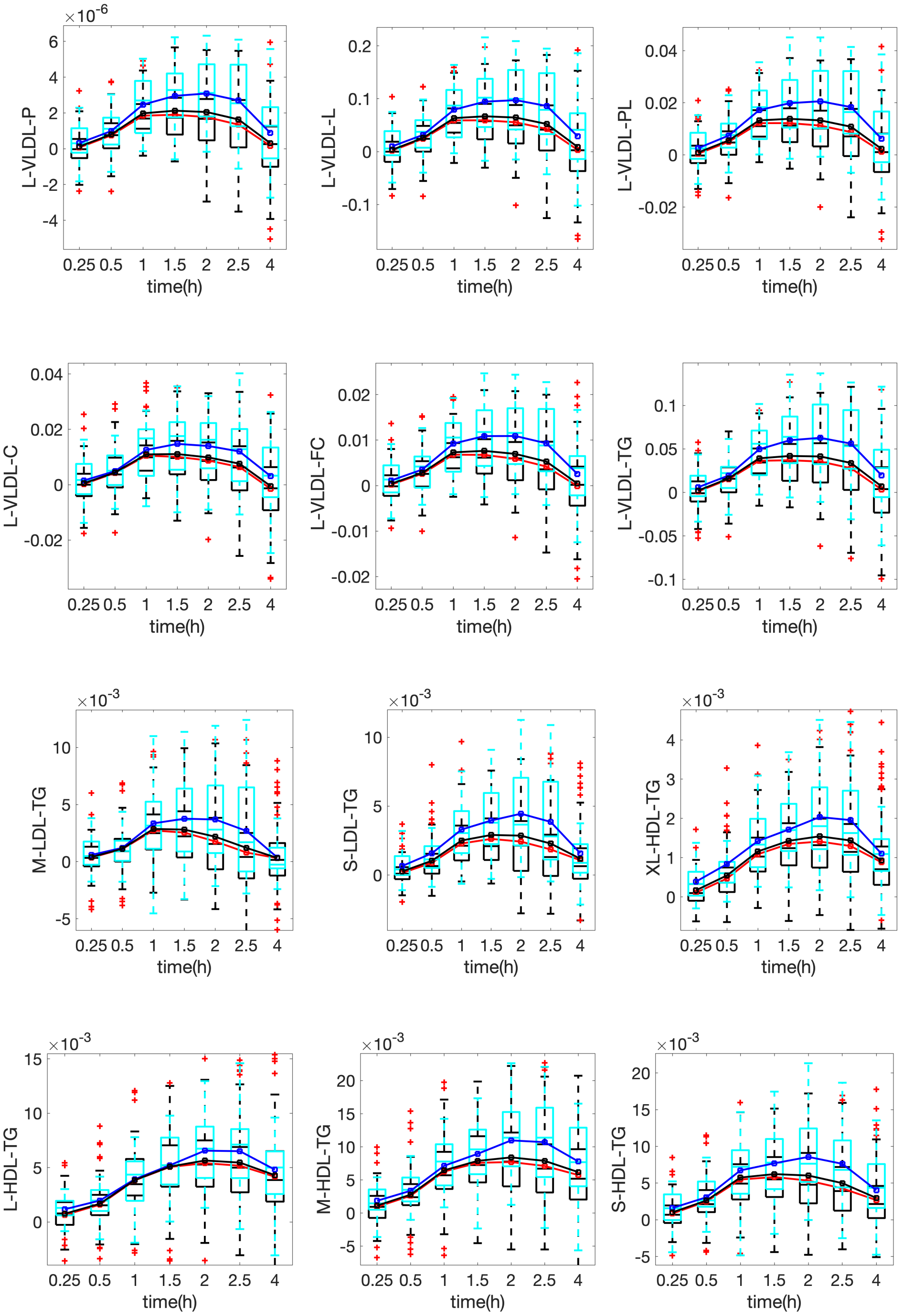

Figure S3.3: Time profiles of selected metabolites (raw data). These belong to the group of metabolites with large absolute coefficients (> 0.1) in $\mathbf{b}_2$ in the 2-component CP model of the T0-corrected data from males.

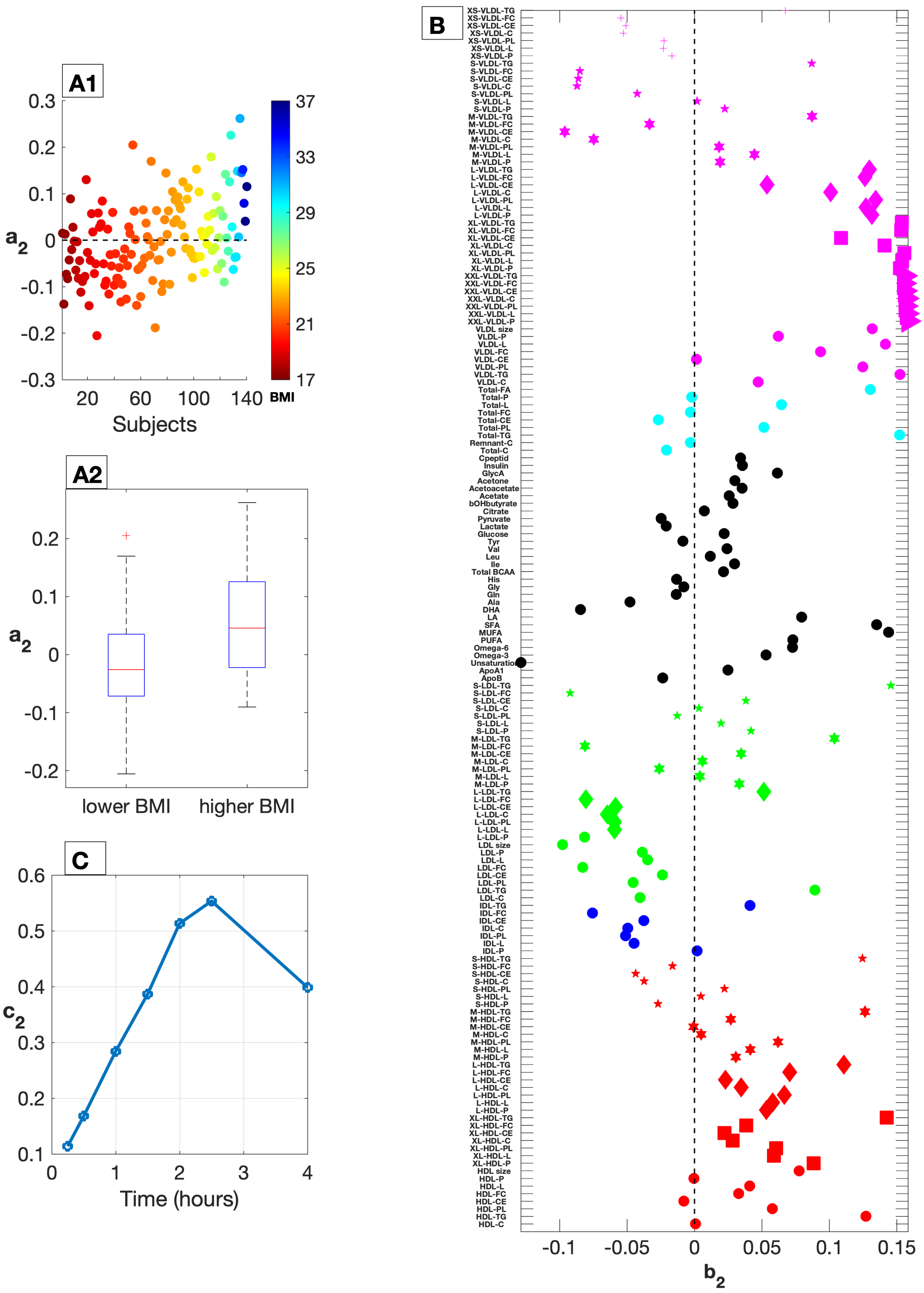

Figure S3.4: The second component, i.e., the component that captures the BMI-related group difference, of the 2-component CP model of the T0-corrected data from males: $\langle \mathbf{a}_2, \mathbf{b}_2, \mathbf{c}_2 \rangle$ are the subjects, metabolites and time factors. Subplots **A1**, **B** and **C** are the factor plots, and subplot **A2** shows the boxplots corresponding to different BMI groups based on the subject scores in $\mathbf{a}_2$. The group difference is statistically significant (p-value $= 6 \times 10^{-4}$).
