# Supplementary material for: Characterizing human postprandial metabolic response using multiway data analysis
Source: Metabolomics. 2024 May 9;20(3):50. doi: 10.1007/s11306-024-02109-y (PMC11082008; doi:10.1007/s11306-024-02109-y)
Supplement: Supplementary file 4 — (pdf 174 KB) [file 11306_2024_2109_MOESM4_ESM.pdf]

# Supplementary Material S4:
# Comparison of CP models from Males vs. Females vs. All Subjects

## 1 CP model of T0-corrected metabolomics data from all subjects

Fig. S4.1 shows the 2-component CP model of the T0-corrected metabolomics data from all subjects, i.e., males and females. The model fit is 45%.

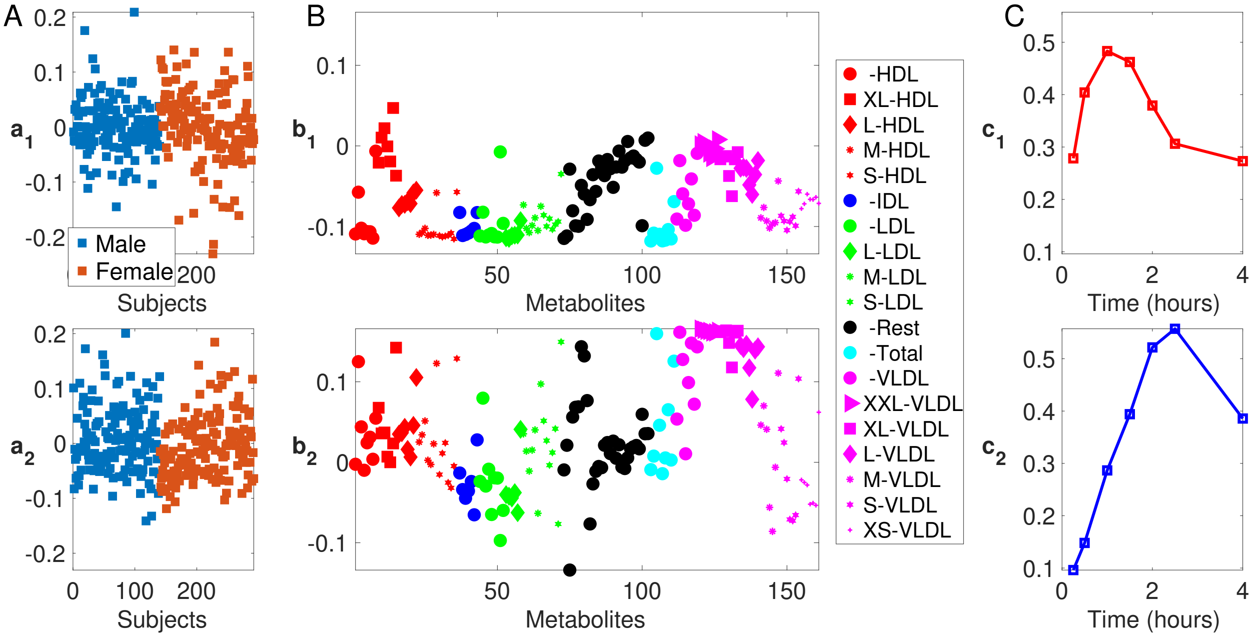


Figure S4.1: 2-component CP model of the T0-corrected metabolomics data from all subjects. (A) *Subjects* mode (i.e., $\mathbf{a}_1$ and $\mathbf{a}_2$). (B) *Metabolites* mode (i.e., $\mathbf{b}_1$ and $\mathbf{b}_2$). (C) *Time* mode (i.e., $\mathbf{c}_1$ and $\mathbf{c}_2$).

The CP model does not reveal any gender-related group difference. The components in the *metabolites* and *time* modes are similar to the components extracted using CP models from only males and only females. This supports our finding that males and females have similar patterns of dynamic response to the challenge test. In the second component, which is similar to the second component of CP models from only males and only females, there is a BMI-related group difference among males (with a $p$-value $= 6 \times 10^{-4}$) but not among females. See the boxplots (using $\mathbf{a}_2$) in Fig. S4.2.

## 2 Patterns of dynamic response to the challenge test are similar in males vs. females vs. all subjects.

Here, we compare the components extracted from the *metabolites* and *time* modes of T0-corrected data using CP models from males vs. females vs. all subjects. Fig. S4.3 shows $\mathbf{b}_1$ and $\mathbf{b}_2$ from the three CP models. We observe that the components from these three CP models are very similar. Fig. S4.4 demonstrates $\mathbf{c}_1$ and $\mathbf{c}_2$ from the three CP models showing that similar temporal patterns are extracted from all data sets.

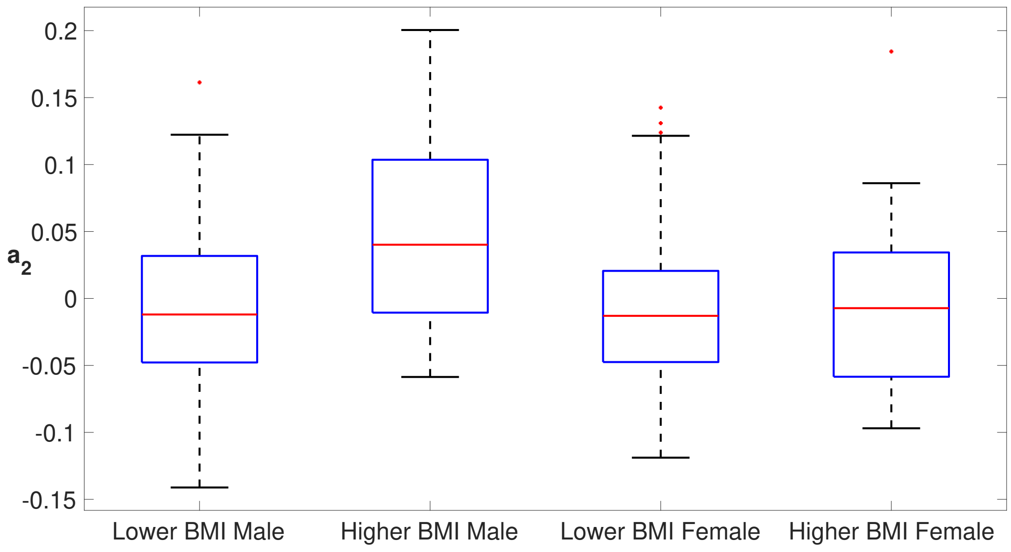


Figure S4.2: Boxplots of $\mathbf{a}_2$ from the 2-component CP model of the T0-corrected data from all subjects.

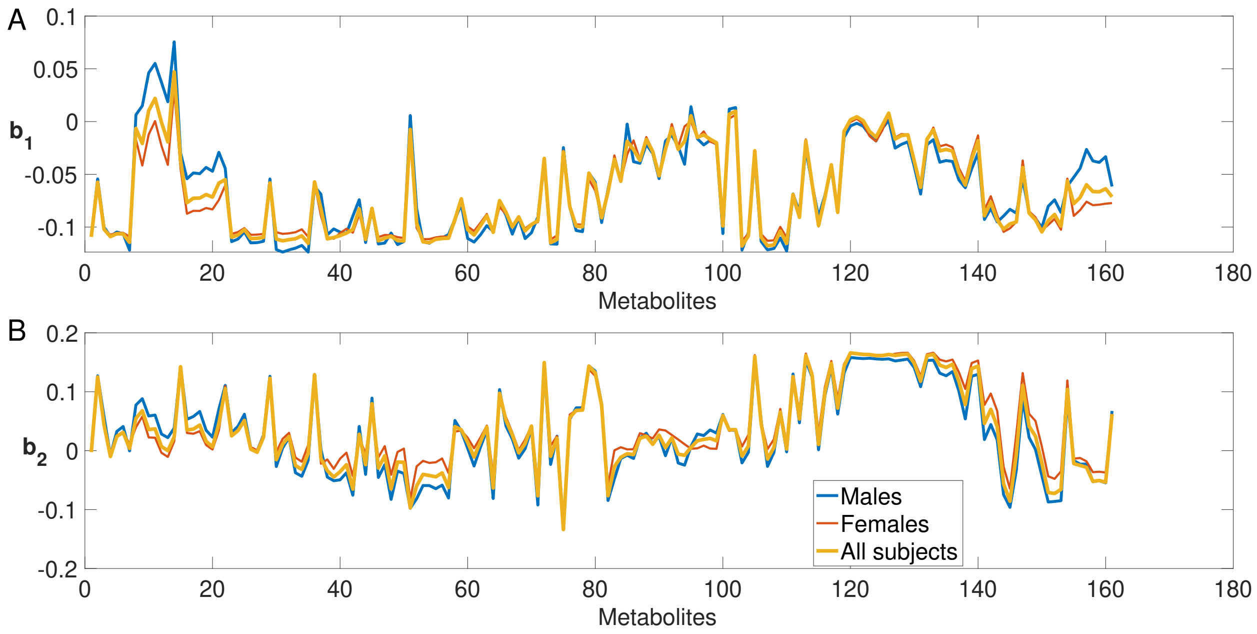


Figure S4.3: Comparisons of the *metabolites* modes using the three CP models from males vs. females vs. all subjects. (A) $\mathbf{b}_1$, (B) $\mathbf{b}_2$.

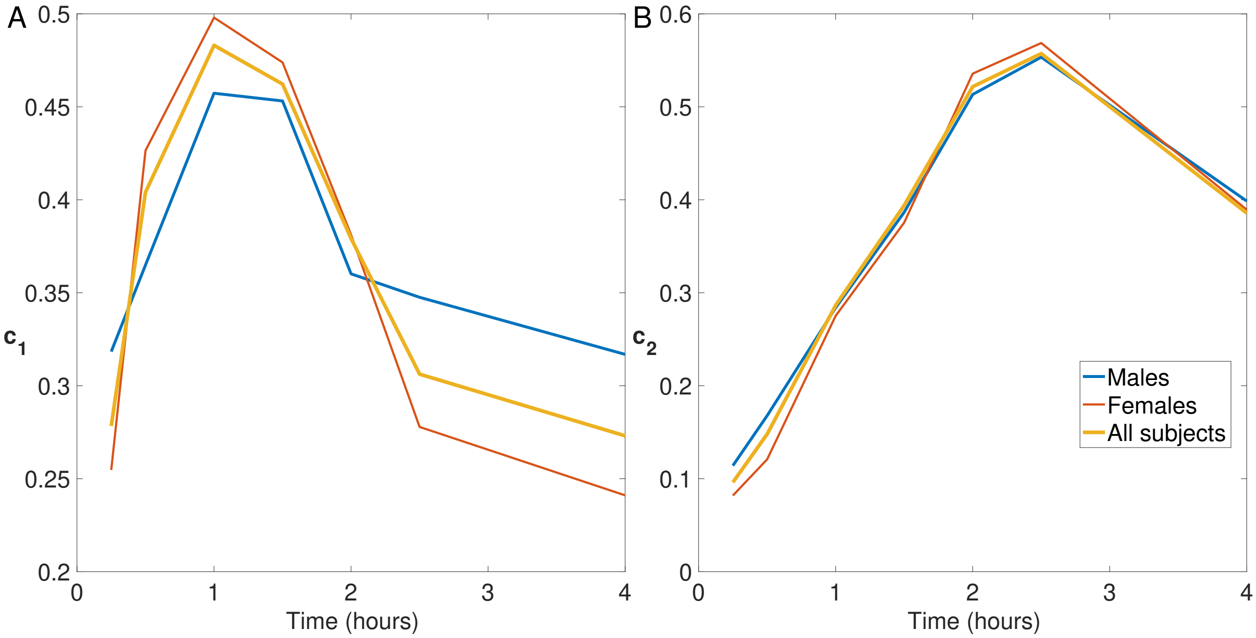


Figure S4.4: Comparisons of the *time* modes using the three CP models from males vs. females vs. all subjects. (A) $\mathbf{c}_1$, (B) $\mathbf{c}_2$.
